# *Bradyrhizobium* Lipid A: Immunological Properties and Molecular Basis of Its Binding to the Myeloid Differentiation Protein-2/Toll-Like Receptor 4 Complex

**DOI:** 10.3389/fimmu.2018.01888

**Published:** 2018-08-14

**Authors:** Luigi Lembo-Fazio, Jean-Marc Billod, Flaviana Di Lorenzo, Ida Paciello, Mateusz Pallach, Sara Vaz-Francisco, Aurora Holgado, Rudi Beyaert, Manuel Fresno, Atsushi Shimoyama, Rosa Lanzetta, Koichi Fukase, Djamel Gully, Eric Giraud, Sonsoles Martín-Santamaría, Maria-Lina Bernardini, Alba Silipo

**Affiliations:** ^1^Dipartimento di Biologia e Biotecnologie “C. Darwin”, Sapienza-Università di Roma, Rome, Italy; ^2^Department of Structural and Chemical Biology, Centro de Investigaciones Biológicas, CIB-CSIC, Madrid, Spain; ^3^Dipartimento di Scienze Chimiche, Complesso Universitario Monte Sant’Angelo, Università di Napoli Federico II, Naples, Italy; ^4^Diomune SL, Parque Científico de Madrid, Madrid, Spain; ^5^Center for Inflammation Research, Unit of Molecular Signal Transduction in Inflammation, VIB, Ghent, Belgium; ^6^Department of Biomedical Molecular Biology, Ghent University, Ghent, Belgium; ^7^Department of Chemistry, Graduate School of Science, Osaka University, Osaka, Japan; ^8^IRD, Laboratoire des Symbioses Tropicales et Méditerranéennes (LSTM), UMR IRD/SupAgro/INRA/UM2/CIRAD, TA-A82/J – Campus de Baillarguet, Montpellier, France; ^9^Istituto Pasteur Italia – Fondazione Cenci Bolognetti, Sapienza-Università di Roma, Rome, Italy

**Keywords:** lipopolysaccharide, innate immunity, inflammatory cytokines, myeloid differentiation protein-2/toll-like receptor 4, *Bradyrhizobium* lipid A, molecular modeling

## Abstract

Lipopolysaccharides (LPS) are potent activator of the innate immune response through the binding to the myeloid differentiation protein-2 (MD-2)/toll-like receptor 4 (TLR4) receptor complexes. Although a variety of LPSs have been characterized so far, a detailed molecular description of the structure–activity relationship of the lipid A part has yet to be clarified. Photosynthetic *Bradyrhizobium* strains, symbiont of *Aeschynomene* legumes, express distinctive LPSs bearing very long-chain fatty acids with a hopanoid moiety covalently linked to the lipid A region. Here, we investigated the immunological properties of LPSs isolated from *Bradyrhizobium* strains on both murine and human immune systems. We found that they exhibit a weak agonistic activity and, more interestingly, a potent inhibitory effect on MD-2/TLR4 activation exerted by toxic enterobacterial LPSs. By applying computational modeling techniques, we also furnished a plausible explanation for the *Bradyrhizobium* LPS inhibitory activity at atomic level, revealing that its uncommon lipid A chemical features could impair the proper formation of the receptorial complex, and/or has a destabilizing effect on the pre-assembled complex itself.

## Introduction

**Figure 1:**
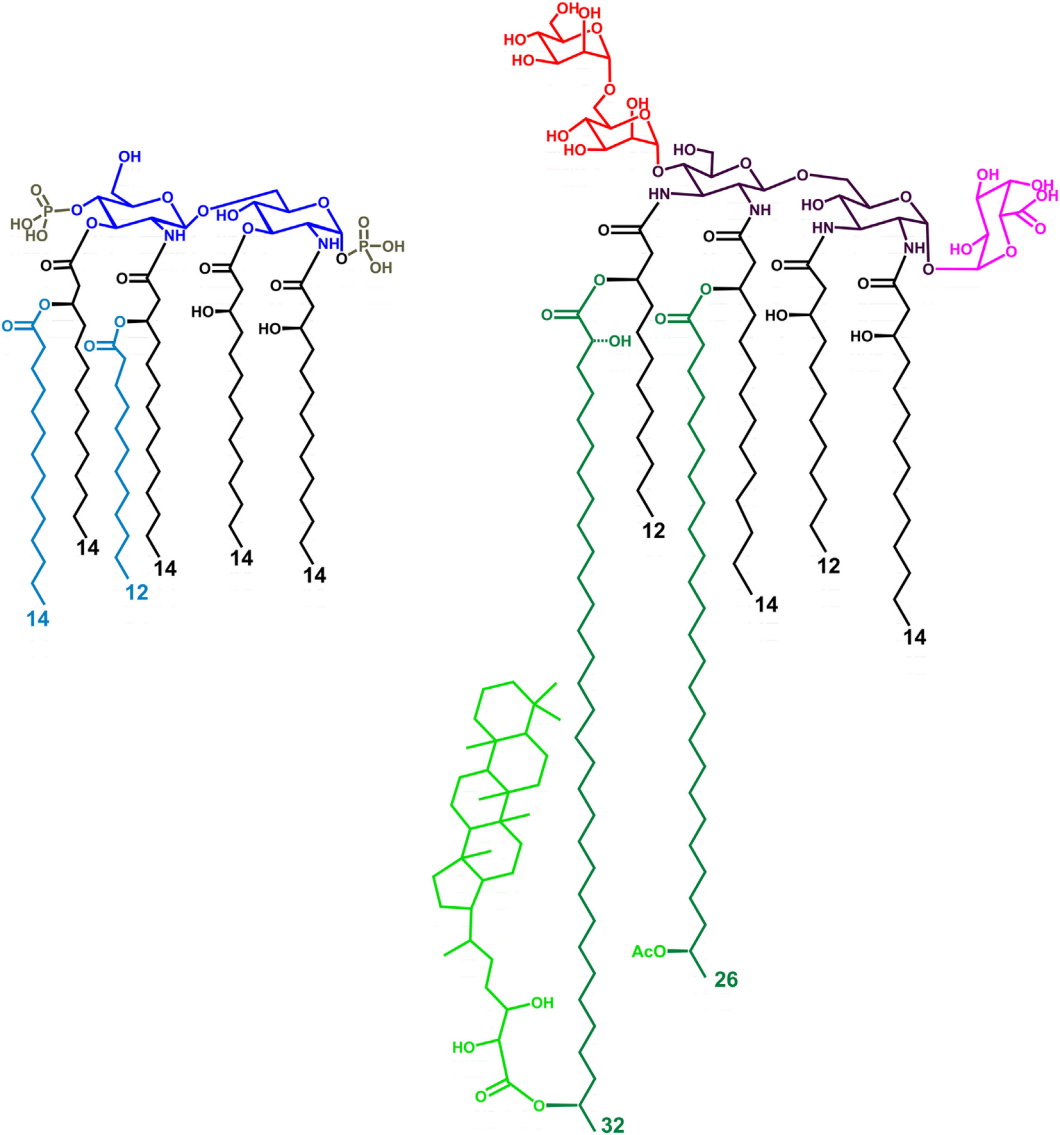
Structure of the LPS lipid A from *Escherichia coli* (left) and *Bradyrhizobium* strains (right, HOLA). The tetra-acylated lipid A from *E. coli* (lipid IV_A_) lacks the two secondary acyl chains (light blue colored in the figure). HF-LA refers to hopanoid-free *Bradyrhizobium* lipid A and is devoid of the Hopanoid moiety (light green). The hexa-acylated lipid A from *E. coli* contains a *bis*-phosphorylated glucosamine disaccharide backbone, asymmetrically substituted by six acyl chains. *Bradyrhizobia* lipid A is mainly constituted of a mixture of hexa- and hepta-acylated species, possesses a 2,3-diaminoglucose (DAG) disaccharide backbone, a galacturonic acid residue on the vicinal DAG and an α-(1→6)-mannose disacchaccaride on the distal DAG. The acyl chains are asymmetrically distributed on the sugar skeleton; of the two secondary very long-chain fatty acids present, one is not stoichiometrically substituted at ω-1 position by a hopanepolyol acid.

Lipopolysaccharides (LPSs) are amphiphilic molecules covering the outer membrane of most Gram-negative bacteria. They are widely known to be involved in the elicitation of immune responses in eukaryotic organisms (1). Structurally, LPSs, in their smooth-form (S-LPS), are tripartite macromolecules built up of a polysaccharide moiety, termed O-antigen, and a core oligosaccharide region covalently linked to a glycolipid domain termed lipid A; the latter is the most conserved part and is responsible for the immunopotency exerted by LPSs isolated from pathogenic Gram-negative bacteria (2). In mammals, the lipid A component of the LPS is the primary immunostimulatory moiety of Gram-negative bacteria and acts as strong stimulator of the innate immunity. The endotoxic properties of the lipid A, namely its capacity to activate the host innate immune response, are strongly influenced by its primary structure. An overacting immune response, due to an uncontrolled and massive circulation of toxic LPS, can result in severe symptoms of sepsis and, in the worst case, septic shock and multi-organ failure. Interestingly, lipid A displaying moderate to low agonist activity can operate as antagonist reducing or, in a dose-dependent manner, completely inhibiting a lipid A’s driven immune activation (1, 3, 4). Lipid A binds the receptor complex made up of toll-like receptor 4 (TLR4) and myeloid differentiation protein-2 (MD-2) on the plasma membrane of immune cells thus activating downstream signaling pathways leading to a rapid release of inflammatory cytokines (1, 5). The highest known immunostimulatory action on human cells is exerted by the *bis*-phosphorylated hexa-acylated lipid A from *Escherichia coli*, characterized by an asymmetric distribution of the acyl chains on the sugar backbone (Figure 1). Structural features such as the nature, number, and distribution of the fatty acid (FA) chains as well as the occurrence of phosphate units, greatly regulate its immunopotency; variation in the acylation or phosphorylation pattern corresponds to a decrease in the immunostimulatory (agonist) activity of the lipid A. One of the first known antagonistic lipid A species acting on the human MD-2/TLR4 complex is a tetra-acylated partial structure termed lipid IV_A_, a biosynthetic precursor of *E. coli* lipid A devoid of the secondary acyl moieties (6). The X-ray crystallographic structure of human MD-2/TLR4 with *E. coli* hexa-acyl LPS (7) provided the molecular basis of lipid A recognition by the MD-2/TLR4 receptor complex. Briefly, five of the six FA chains of *E. coli* LPS are buried inside the lipophilic pocket of the MD-2 protein whereas the sixth acyl chain is partially extruded, lying on the surface and interacting with the partner TLR4 (dubbed TLR4*). This interaction is the basis for receptor complex dimerization, promoting the intracellular juxtaposition of the TIR domains and leading to signal transduction culminating in the elicitation of the inflammatory process. The X-ray crystallographic structure of the tetra-acylated lipid IV_A_ in complex with human MD-2/TLR4 receptor demonstrated that all four acyl chains are sitting inside the MD-2 binding pocket in a fashion that does not allow dimerization and subsequent activation (1, 8).

Noticeable efforts have been made so far to identify natural or *ad hoc* synthesized lipid A significantly different from enterobacterial counterparts and able to interfere or modulate immune/inflammatory responses mediated by toxic lipid A, such as lipid IV_A_ and Eritoran (1, 9). These lipid A variants are the most studied ligands of MD-2/TLR4, which is considered a molecular target related to several inflammatory pathologies but also to “modern-day” diseases, including allergies, asthma, and autoimmune disorders (10). However, the natural repertoire of LPSs is undeniably much more diverse, thus opening the possibility to find natural compounds to active as TLR4 immunomodulators or alternatively, to synthesize bio-inspired lipid A derivatives in a perspective of therapy-related drug design.

In this context, rhizobial lipid A remarkably differs from enterobacterial analogs in terms of acylation and phosphorylation pattern as well as in the sugar backbone. Previous studies reported indeed the weak endotoxic activity of rhizobial lipid A as well as its inhibitory properties toward the toxic effects of enterobacterial LPS (11–14), with the single exception of *S. meliloti* (15). Rhizobia are Gram-negative bacteria able to establish symbiotic relationship with legumes and to reduce atmospheric nitrogen into ammonium, thus providing nitrogen nutrition to the host plants (16, 17). Rhizobia belonging to the *Bradyrhizobium* genus are the most commonly found symbiont for most legume species in habitats worldwide and constitute the most commonly used inoculants for cultivated plants of first agronomic importance (as soybean, peanut, and cowpea) (18–20). *Bradyrhizobium* lipid A (21–24) (Figure 1), included the strains ORS278 and ORS285 here used (25), comprises a mixture of species differing by the number, length, and nature of the acyl chains characterized by (i) a pentasaccharide sugar backbone; (ii) the occurrence of very long-chain fatty acids (VLCFA), which have been demonstrated to be pivotal in the bacterium adaptation to intracellular life (26–29); and (iii) a hopanoid moiety covalently linked to the VLCFA, present in a non-stoichiometric fashion.

The observation of expressing the unusual lipid A structure of Bradyrhizobia strains prompted us to evaluate the impact of *Bradyrhizobium* LPS/lipid A on the innate immune system. The results reported herein revealed an extremely low capability to elicit an immune response when tested on both murine and human cells. More intriguingly, a strong inhibitory activity toward the potent agonist *E. coli* LPS was observed. In order to shed light on this behavior, we also investigated the molecular basis of the mechanism of *Bradyrhizobium* LPS/lipid A binding to the human MD-2/TLR4 complex by computational approach, with the aim to provide a molecular model explaining the antagonist properties of *Bradyrhizobium* lipid A.

With this work we meant to complement and expand previous studies highlighting the potential of rhizobia LPS as a promising natural source of MD-2/TLR4 immunomodulatory compounds, that can be of inspiration for development of vaccine adjuvants and/or endotoxin-based therapeutics as an alternative approach in the treatment of inflammatory disorders.

## Materials and Methods

### HEK 293 hTLR4/CD14/MD2 Cell Culture, Transfection, and Stimulation

HEK293 cell line, stably transfected with human TLR4/MD2-CD14 (InvivoGen) was seeded into 96-well plate at the concentration of 1 × 10^5^ cells/mL. 48 h after seeding the cells were transiently transfected through PolyFect Transfection Reagent (Qiagen) with a reaction mix containing 150 ng of Firefly luciferase reporter constructs, pGL3.ELAM.tk [harboring nuclear factor kappa B (NF-κB) promoter sequences], and 15 ng of *Renilla* luciferase reporter plasmid, pRLTK (as an internal control). The day after the cells were incubated with different concentrations of *Bradyrhizobium* lipid A or LPS [1, 10, and 100 ng/mL; the LPS and the lipid A preparations were obtained as previously described (21–24)], or with purified *E. coli* LPS (LPS-EB ultrapure; InvivoGen) or with synthetic lipid IV_A_ used at the same concentrations as above, for 6 h to analyze NF-κB activity (Dual Luciferase Reporter Assay System, Promega) and to measure CXCL-8 release (DuoSet R&D System). For the competition assay, HEK 293-TLR4/MD2-CD14 cells were primed with 1, 10, and 100 ng/mL of *Bradyrhizobium* LPS or lipid A, or lipid IV_A_ or with *Shigella flexneri* hexa-acylated LPS for 1 h and then exposed to *E. coli* LPS (10 and 100 ng/mL) for 4 h (30). After this time, NF-κB activity and CXCL-8 production were measured.

### BMDMs Isolation, Culture, and Stimulation

C57BL/6 mice were purchased from Charles River (Charles River ITALY). BMDMs were derived from the bone marrow cells collected from 5-week-old female mice, as already reported (31). Animal studies were conducted according to protocols approved by the University of Rome La Sapienza and adhered strictly to the Italian Ministry of Health guidelines for the use and care of experimental animals.

BMDMs were differentiated during 7 days in RPMI 1640 (Lonza, Italy), supplemented with 10% of heat-inactivated FBS (HycloneTM, Euroclone, Italy), 1% di l-glutamine (Lonza, Italy), 1% sodium pyruvate (Lonza, Italy), 1% NEAA (Lonza, Italy), 0.5% 2-ME (Gibco, Italy), and 40 ng/mL macrophage colony-stimulating factor (M-CSF; Miltenyi Biotec). BMDMs were seeded into 24-well plate (5 × 10^5^ cells per well) and were incubated with different concentrations of *Bradyrhizobium* LPS or lipid A (1, 10, or 100 ng/mL) or with *E. coli* LPS or with lipid IV_A_ at the same concentrations as above for 6 h. Where necessary, polyinosinic-polycytidylic acid, Poly (I:C) (Invivogen) was used at the concentration of 5 µg/mL. After this time the supernatants were collected and tumor necrosis factor TNF release was measured through ELISA (DuoSet R&D System). For the competition assay, BMDMs were pre-incubated with *Bradyrhizobium* LPS or lipid A (10, or 100 ng/mL) or with lipid IV_A_ at the same concentrations as above for 1 h and then exposed to *E. coli* LPS (10 ng/mL) for 4 h. After this time, TNF and CXCL-1 release was quantified *via* ELISA.

### RNA Extraction and *q*PCR Analysis of *ifn*-β Expression

Total RNA was extracted from unstimulated or LPS or treated or Poly (I:C)-treated C57BL/6 BMDMs through Trizol reagent (Invitrogen) according to the manufacturer’s instructions. RNA was converted to cDNA using High Capacity cDNA Archive kit (Applied Biosystems, Monza, Italy) and random primers, and finally amplified using Power SYBR 17 Green PCR Master Mix (Applied Biosystem,). The 2^−ΔΔCt^ method was applied to analyze the relative changes in expression profiling of interest genes, as already reported (32). Values were normalized to the internal *tbp* gene control. Primers for *q*PCR are for *ifn*-β: F-TCCGAGCAGAGATCTTCAGGAA; R-TGCAACCACCACTCATTCTGAG. For *tbp*: F-CTG GAA TTG TAC CGC AGC TT; R-TCC TGT GCA CAC CAT TTT TC.

### Human Peripheral Blood Mononuclear Cells (PBMCs) Isolation and Stimulation

Peripheral blood mononuclear cells were isolated from buffy coats obtained by the blood bank of Sapienza University from healthy adult volunteers (blood donors) following written informed consent. CD14+ monocytes were isolated from PBMCs using the MACS system (Miltenyi Biotec, Bergisch Gladbach, Germany) and cultured in complete RPMI 1640 medium (Lonza, Italy, Milan), supplemented with 10% of heat-inactivated FBS (HycloneTM, Euroclone, Italy, Milan), 1% di l-glutammine (Lonza), 1% of non-essential amino acid solution (NEEA—Lonza), 1% sodium pyruvate (Lonza), penicillin 100 U/mL–streptomycin 100 µg/mL (Lonza), and 0.1% di 2-ME (Gibco, Italy). CD14+ monocytes were seeded at the concentration of 5 × 10^5^ cells/well in 12-multiwell plate and exposed to 0.05, 0.5, 1, and 10 ng/mL of LPS or lipid A from *Bradyrhizobium* or to commercial *E. coli* O111:B4 or to synthetic lipid IV_A_ at same concentrations as above for 12 h. Cell supernatants were then collected and processed for ELISA to measure the levels of TNF and IL-6.

### Cytokine Measurement

Murine and human cytokines were determined in supernatants of stimulated cells by using R&D Systems DuoSet ELISA kits according to the manufacturers’ instructions.

### Molecular Modeling

#### Structure Construction

The 3D structures of both the hopanoid-containing and HOLA and HF-LA were built with PyMOL molecular graphics and modeling package based on the saccharide backbone of *E. coli* LPS retrieved from the PDB ID 3FXI. Atoms were modified and added accordingly and bond type and length were carefully selected and revised. The geometry of these two structures was further optimized with Maestro under the OPLS3 force field. The antagonist conformation of the hTLR4/MD-2 complex was assembled by merging the ectodomain of TLR4 from RCSB (www.rcsb.org) PDB ID 3FXI and MD-2 from PDB ID 2E59. The latter was aligned to the spatial coordinates of the MD-2 present in 3FXI and solvent, ligands, and ions were removed.

#### Structure Optimization

Hydrogen atoms were added to the X-ray structures using the preprocessing tool of the Protein Preparation Wizard of the Maestro package, and then the structures went through a restrained minimization under the OPLS3 force field with a convergence parameter to RMSD for heavy atoms kept default at 0.3 Å.

#### Docking Procedure

Gasteiger charges were computed and assigned with AutoDockTools 1.5.6 to both the proteins and the ligands. Both HOLA and HF-LA were left flexible by allowing some appropriately selected dihedral angles to rotate whereas the receptor was always kept completely rigid. The docking was performed with AutoDock Vina (33). A cubic docking box of 60 Å in size and 1 Å in spacing was defined. The box was centered equidistant to the geometric center of residues Arg90 (MD-2), Arg96 (MD-2), and Arg264 (TLR4).

#### Parameterization

The full lipid structures were split into residues to facilitate and homogenize the parameterization process. The partial charges and atom types of the 4-substituted and the 6-substituted 2,3-diamino-2,3-dideoxy-glucose (DAG) monosaccharides composing the oligosaccharide backbone were established respectively based on residues 4YB (4-substituted GlcNAc) and 6YA (6-substituted GlcNAc) of the GLYCAM force field (34). The partial charges and parameters for the two mannose (Man) residues and the galacturonic acid (GalA) were retrieved from the GLYCAM force field respectively under the name 0MA, 6MA, and 1OA. Partial charges for the primary and secondary acylation as well as for the hopanoid residue were derived, with the help of antechamber (31), following the standard GAFF procedure described in the AMBER manual and the parameters were assigned by the GAFF force field.

#### MD Simulations

All MD simulations were performed with AMBER14 (35), the protein was described by the ff14SB all-atom force field (36), the pentasaccharide backbone of the BTAi1 lipid A by the GLYCAM_06j-1 force field (34) and the other constituents of the lipid A (the lipid chains and the hopanoid moiety) were parameterized with the General Amber Force Field (GAFF) (37). The simulation box was designed such as the edges are distant of at least 10 Å of any atoms. The system was solvated with the TIP3P water molecules model. One Na^+^ ion was added to counterbalance the negative charge of the galacturonate group. All the simulations were performed with the same equilibration and production protocol. The equilibration protocol contains height sequential steps. The first one consists of 1,000 steps of steepest descent algorithm followed by 7,000 steps of conjugate gradient algorithm; a 100 kcal mol^−1^.Å^−2^ harmonic potential constraint is applied on both the proteins and the ligand. In the fourth subsequent steps, the harmonic potential is progressively lowered (respectively to 10, 5, 2.5, and 0 kcal mol^−1^.Å^−2^) for 600 steps of conjugate gradient algorithm each one. In the sixth step, the system is heated from 0–100 K by a Langevin thermostat in the canonical ensemble (NVT) under a 20 kcal mol^−1^.Å^−2^ harmonic potential restraint on the proteins and the ligands. The next step heats up the system from 100–300 K in the isothermal-isobaric ensemble (NPT) under the same restraint condition than the previous step. In the last step, the same parameters are used to simulate the system for 100 ps but no harmonic restraint is applied. At this point, the system is ready for the production run, which is performed in the NPT ensemble.

## Results

### Immunomodulatory Properties of *Bradyrhizobium* LPS and Lipid A

#### Immunological Tests of *Bradyrhizobium* LPS and Lipid A on HEK293 hTLR4 Cell Line

**Figure 2:**
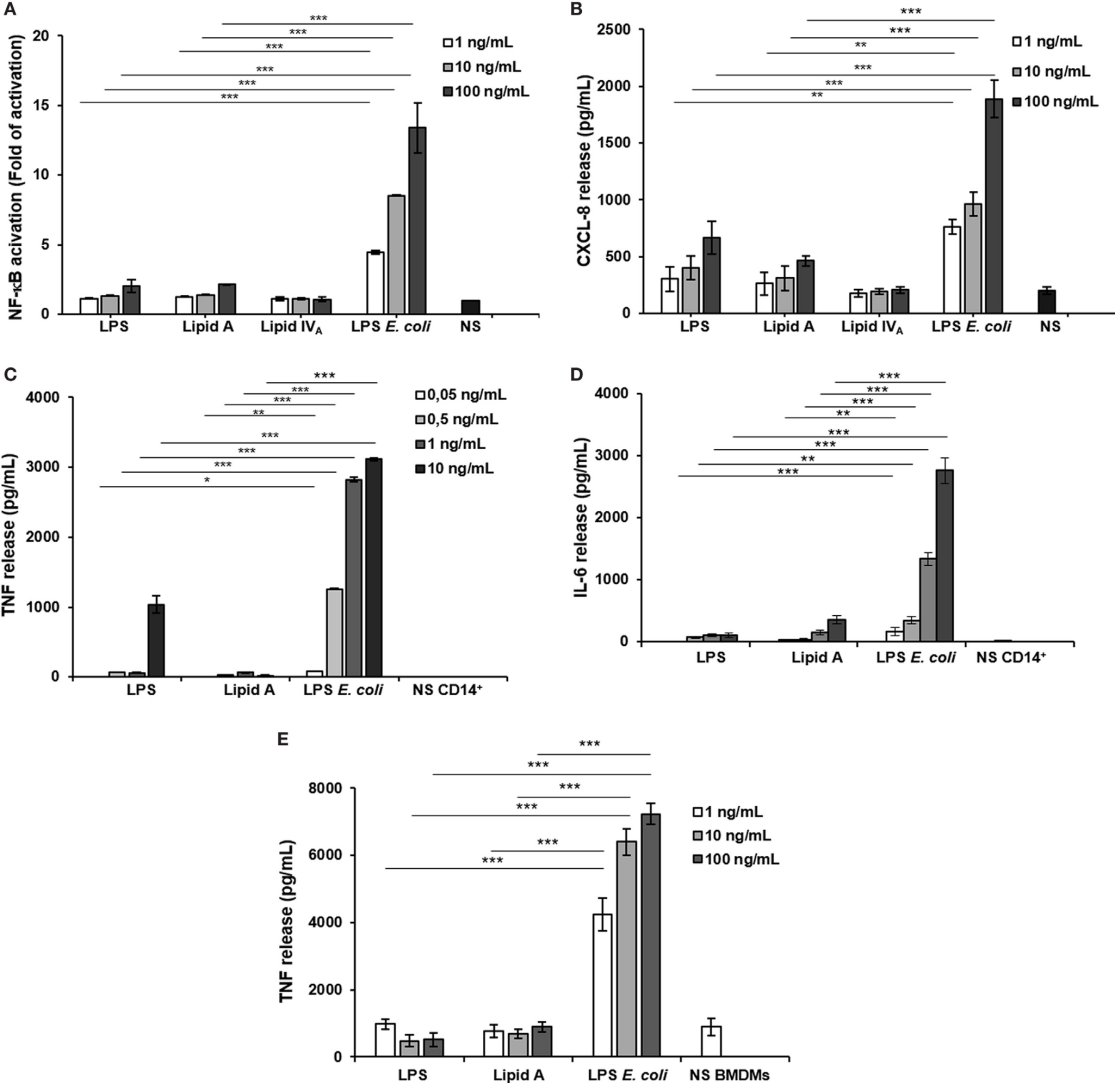
Analysis of immunopotential of *Bradyrhizobium* LPS/lipid A in HEK293 hTLR4 cell line, monocytes, and BMDMs. Activation of nuclear factor kappa B **(A)** and CXCL-8 production **(B)** in HEK 293 hTLR4/MD2-CD14 stimulated with *Bradyrhizobium* LPS/lipid A at the concentration of 1, 10, and 100 mg/mL for 6 h. Commercial hexa-acylated *Escherichia coli* LPS or synthetic lipid IV_A_ were used at the same concentrations as positive and negative control, respectively. tumor necrosis factor TNF **(C)** and IL-6 **(D)** release in cell-free supernatants of monocytes stimulated with *Bradyrhizobium* LPS/lipid A at the concentration of 0.05, 0.5, 1, and 10 mg/mL for 12 h. Commercial hexa-acylated *E. coli* LPS was used at the same concentrations as control. TNF **(E)** production in BMDMs stimulated with *Bradyrhizobium* LPS/lipid A at the concentration of 1, 10, and 100 mg/mL for 6 h. Abbreviation: NS: not stimulated. Data are expressed as mean ± SD of three independent experiments in triplicate. *p < 0.05, **p < 0.01, and ***p < 0.001, after Student’s *t*-test.

To evaluate the immunological impact of *Bradyrhizobium* LPS and eventually lipid A, we first analyzed their effect at different concentrations (1, 10, and 100 ng/mL) in the HEK293 cell line (38), stably transfected with human CD14/MD-2/TLR4. Similar concentration of the hexa-acylated, highly stimulatory LPS of *E. coli* O111:B4 and the synthetic lipid IV_A_ were used as agonistic and antagonistic controls, respectively. Likewise, untreated cells were considered as the negative control in all the experiments shown. Stimulation of HEK293 hTLR4 was carried out for 6 h (Figures 2A,B).

With a parallel approach, we used HEK293 hTLR2 cells to assess whether the biological activity of *Bradyrhizobium* LPS could be extended to TLR2, in addition to TLR4 as described for a limited number of LPS (Figure S1A in Supplementary Material) (39). Furthermore, assessment of the *Bradyrhizobium* LPS in this cell model could rule out a potential role for lipopeptides L(Ps) contaminating LPS. In HEK293 hTLR2 cells, Pam3CSK4 (Pam3) (500 ng/mL), a synthetic triacylated lipopeptide (LP) that mimics the acylated amino terminus of bacterial LP, represented the positive control for a TLR2 ligand. Cells treated with *E. coli* LPS, as above, or untreated cells were used as the negative controls. Activation of NF-κB and Chemokine (C-X-C motif) ligand 8 (CXCL-8, also known as IL-8) production was measured after 6 h of stimulation in both assays. As shown in Figures 2A,B, stimulation of HEK293 hTLR4 with *Bradyrhizobium* LPS and lipid A stimulation elicited a poor NF-κB activation and CXCL-8 production, which were significantly lower with respect to the values induced by *E. coli* LPS stimulation (for NF-κB and CXCL-8, *Bradyrhizobium* LPS at 10 and 100 ng/mL vs. *E. coli* LPS at same concentrations *p* < 0.001). No activation of NF-κB and CXCL-8 production (Figures S1A,B in Supplementary Material) were observed upon HEK293 hTLR2 stimulation with *Bradyrhizobium* LPS and lipid A, suggesting that neither LPS nor lipid A could trigger the TLR2-mediated signaling.

#### Immunological Impact of *Bradyrhizobium* LPS and Lipid A on CD14^+^ Derived Monocytes Derived From PBMCs

The low immunological impact of *Bradyrhizobium* LPS and lipid A was also confirmed in human CD14+ derived monocytes derived from PBMCs, which express all TLRs. Cells were exposed to *Bradyrhizobium* LPS and lipid A at the concentration of 0.05, 0.5, 1, and 10 ng/mL for 12 h. *E. coli* LPS was tested in parallel at same concentrations. The release of the pro-inflammatory cytokines TNF and interleukin-6 (IL-6) was assessed as readout. The results are shown in Figures 2C,D. In agreement to what observed in the HEK293 hTLR4 cell model, with *Bradyrhizobium* lipid A the release of these cytokines was poorly detectable at any concentration tested while 10 ng/mL *Bradyrhizobium* LPS clearly induced TNF and IL-6, albeit both were significantly lower than those elicited by *E. coli* LPS at the same concentration (for TNF, *Bradyrhizobium* LPS at 1 and 10 ng/mL vs. *E. coli* at same LPS concentration *p* < 0.001; for IL-6, all *Bradyrhizobium* LPS concentrations vs. *E. coli* all concentrations *p* < 0.001).

#### Immunological Impact of *Bradyrhizobium* LPS and Lipid A on BMDMs From C57BL/6 Cells

To strengthen the results obtained in the HEK293 cell models and PBMCs, we also used BMDMs from C57BL/6 wild-type mice or from C57BL/6 knockout mice for *Tlr2* (*Tlr2*−/−) or *Tlr4* (*Tlr4*−/−) (Figures S1C,D in Supplementary Material). Murine macrophages were exposed to *Bradyrhizobium* LPS and lipid A at 1, 10, and 100 ng/mL. Pam3 (1 µg/mL) and *E. coli* LPS at the same concentrations as above, were the positive controls for TLR2 and TLR4, respectively. After 6 h stimulation, TNF production was quantified. In wild-type macrophages, stimulation with either *Bradyrhizobium* LPS or lipid A induced significantly less TNF compared to stimulation with Pam3 (all *Bradyrhizobium* LPS concentrations vs. *E. coli* all concentrations *p* < 0.001) as shown in Figure 2E. As observed in PBMCs, *Bradyrhizobium* LPS and lipid A induced a scantly amount of this cytokine (all *Bradyrhizobium* LPS and lipid A concentrations vs. *E. coli* all concentrations *p* < 0.001).

When the same experimental set-up was applied to murine macrophages of *tlr2*−/− or *tlr4*−/− mice, we found that the absence of *tlr2* prevents the production of TNF upon Pam3CSK stimulation but not following LPSs and lipid A stimulation, as expected. On the contrary, in *tlr4*−/− mice TNF production was null and high following LPSs/lipid A and Pam3 stimulation, respectively (Figures S1C,D in Supplementary Material). These results confirm those achieved in the HEK293 cell models: i.e., the reduced immunostimulatory of *Bradyrhizobium* LPS and lipid A is strictly dependent on TLR4 and not on TLR2.

**Figure 3:**
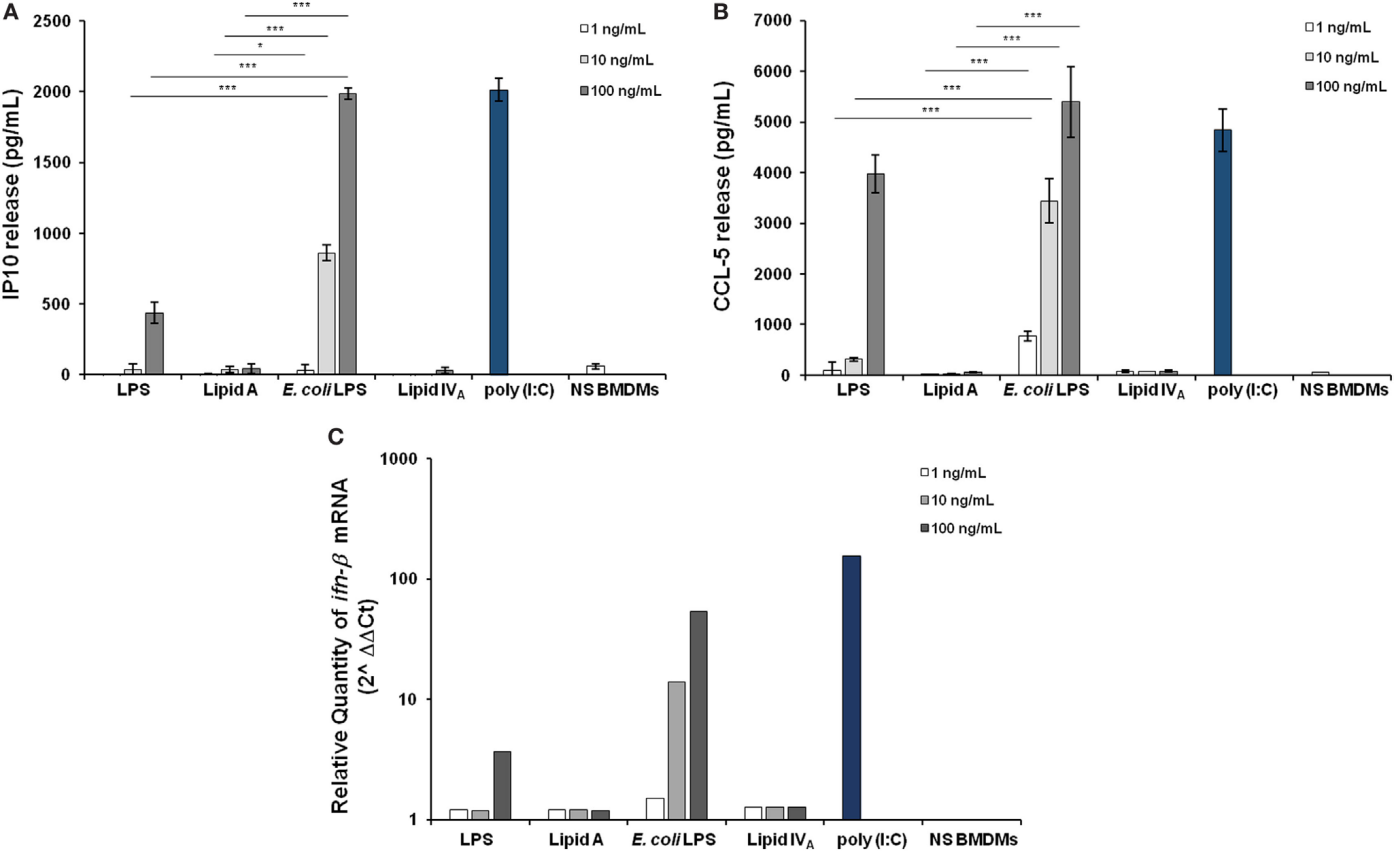
Assessment of the involvement of Trif pathway upon stimulation of BMDMs with *Bradyrhizobium* LPS/lipid A. Inducible protein 10 **(A)** and CCL-5 production **(B)** in BMDMs stimulated with *Bradyrhizobium* LPS/lipid A at the concentration of 1, 10, and 100 ng/mL for 6 h. Commercial hexa-acylated *Escherichia coli* LPS or synthetic lipid IV_A_ were used at the same concentrations. The TLR3 agonist poly (I:C) (5 µg/mL) was used as a positive control of Trif pathway activation. Data are expressed as mean ± SD of three independent experiments in triplicate. *p < 0.05, **p < 0.01, and ***p < 0.001, after Student’s *t*-test. **(C)** *q*PCR of *ifn-*β mRNA following 3 h of stimulation of BMDMs with LPS/lipid A at the same concentrations, as above. Results are normalized to the internal TBP gene control and are presented on a logarithmic scale as the ratio of gene expression between stimulated and unstimulated BMDMs.

Downstream TLR4-mediated LPS signaling two pathways could be activated. The adaptor protein MyD88 engages a pathway leading to the production of pro-inflammatory cytokines, such as TNF and CXCL-1. The other pathway involves the proteins Mal (also called TIRAP), TRIF, and TRAM, allowing the expression of genes encoding type 1 interferon and interferon-associated genes (40, 41). Therefore, we faced the question of whether *Bradyrhizobium* LPS and/or lipid A could activate the Trif pathway. With this aim, BMDMs were stimulated as above with 1, 10, or 100 ng/mL of *Bradyrhizobium* lipid A or LPS. *E. coli* LPS and lipid IV_A_ were used at same concentrations as a control. Polyinosinic-polycytidylic acid, Poly (I:C)—a synthetic analog of double stranded RNA—which acts as TLR3 agonist was used at the concentration of 5 µg/mL to elicit the Trif pathway. After 6 h of stimulation, we measured the release of the cytokine interferon inducible protein 10 (IP-10), which is induced by the axis LPS-TRIF-IRF3-IFN (42) and the chemokine RANTES (CCL-5,) which is expressed through the involvement of IRF3 (43, 44). *Bradyrhizobium* lipid A and lipid IV_A_ did not elicit any IP-10 release while *Bradyrhizobium* LPS at the concentration of 100 ng/mL could induce a low production of IP-10, which was significantly lower than those observed with *E. coli* LPS at the concentrations of 10 and 100 ng/mL and Poly (I:C) (for both *p* < 0.001), as shown in Figure 3A. The same trend was observed for CCL-5 (Figure 3B). As IFN-β release can be undetectable upon BMDM stimulation with the fully immunocompetent hexa-acylated *E. coli* LPS (30) we directly proceeded to analyze the mRNA levels for this cytokine. BMDMs were stimulated with 1, 10, or 100 ng/mL of *Bradyrhizobium* LPS or lipid A or with *E. coli* or with lipid IV_A_ at the same concentrations. Poly (I:C) was used as above. After 3 h of stimulations BMDMs were processed to measure the levels of *ifn*-β RNA through *q*PCR. In accordance with results of IP-10 and CCL-5 release, *ifn*-β RNA was undetectable with *Bradyrhizobium* lipid A while *Bradyrhizobium* LPS at the concentration of 100 ng/mL could trigger a low expression of *ifn*-β (Figure 3C). As expected, *E. coli* LPS and Poly (I:C) determined high levels of *ifn*-β RNA. Definitely, these data suggest that *Bradyrhizobium* LPS is a poor elicitor of the Trif pathway.

#### Competition Tests of *Bradyrhizobium* LPS and Lipid A on HEK293 hTLR4 and on C57BL/6 BMDMs Cell Lines

**Figure 4:**
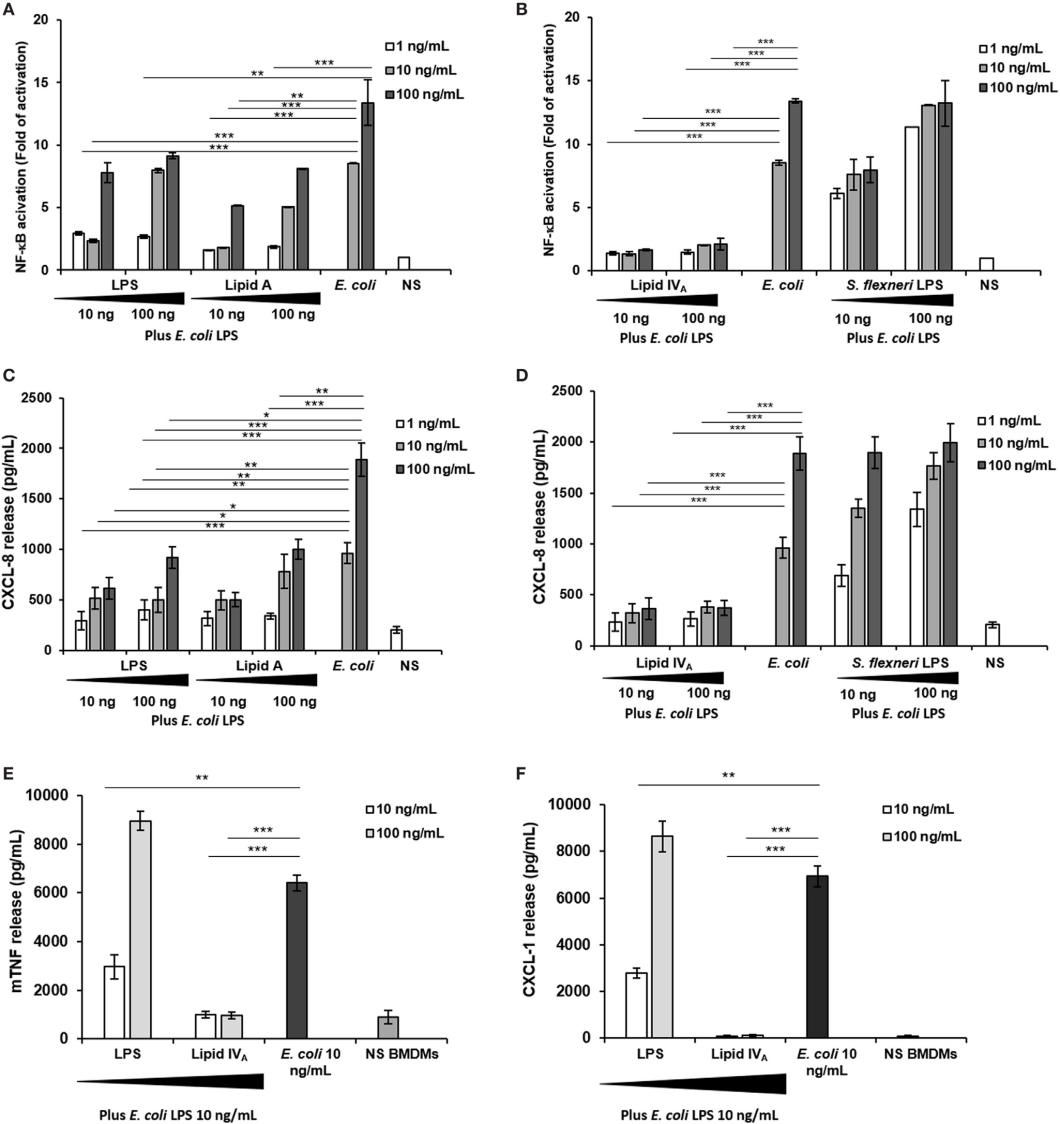
Competition assay: **(A–D)** HEK293 hTLR4 cell line; **(E,F)** BMDMs. **(A,B)** Fold of nuclear factor kappa B activation and **(C,D)** CXCL-8 release following priming with 10 and 100 ng/mL of *Bradyrhizobium* LPS or lipid A, or with lipid IV_A_ or with *Shigella flexneri* hexa-acylated LPS for 1 h and then exposed to *Escherichia coli* LPS (10 and 100 ng/mL) for 4 h. Stimulation with the only *E. coli* LPS (10 and 100 ng/mL) for 5 h was used as a control. **(E,F)** BMDMs were stimulated with 10 and 100 ng/mL of *Bradyrhizobium* LPS or with lipid IV_A_ during 1 h and then incubated with 10 ng/mL of *E. coli* LPS for 4 h. After this time TNF and CXCL-1 release were quantified. Stimulation with the only *E. coli* LPS (10 ng/mL) for 5 h was used as a control. Data are expressed as mean ± SD of three independent experiments in triplicate. *p < 0.05, **p < 0.01, and ***p < 0.001, after Student’s *t*-test.

Finally, as some under-acylated lipid A (45) and, more recently, unusual LPS-containing long FA chains (46), have been reported to show an inhibitory activity against endotoxically active LPS (47), we assessed the ability of *Bradyrhizobium* LPS to affect the TLR4-mediated signaling triggered by the hexa-acylated, fully immunocompetent *E. coli* LPS. With this aim, HEK 293 hTLR4 cells were pre-incubated for 1 h with *Bradyrhizobium* LPS and stimulated with 10 and 100 ng/mL of *E. coli* LPS for 4 h (Figures 4A–D). NF-κB activation and CXCL-8 production were quantified after this time. The synthetic tetra-acylated lipid IV_A_ was used in parallel under the same conditions as for *Bradyrhizobium* LPS as a control of the inhibitory effect. A hexa-acylated LPS of *Shigella flexneri* (30) was used in parallel. Untreated cells and cells stimulated with the *E. coli* LPS at 10 and 100 ng/mL for 4 h were the control in this experiment. Under these experimental conditions, *Bradyrhizobium* LPS showed an inhibitory activity on *E. coli* LPS at all the concentrations tested (NF-κB values of *Bradyrhizobium* LPS + *E. coli* LPS vs. *E. coli* LPS alone, for NF-κB; *Bradyrhizobium* LPS at 1 and 10 ng/mL *p* < 0.001; *Bradyrhizobium* LPS at of 100 ng/mL *p* < 0.01).

Finally, we evaluated whether *Bradyrhizobium* LPS could also interfere with TLR4 signaling in murine cells which have been reported to be less sensitive to the degree of lipid A acylation (48, 49). Therefore, BMDMs were stimulated with 10 and 100 ng/mL of *Bradyrhizobium* LPS for 1 h and then exposed to 10 ng/mL of *E. coli* LPS for 4 h (Figures 4E,F). Unstimulated cells and BMDMs stimulated with 10 ng/mL *E. coli* LPS for 5 h were the controls. The production of TNF and CXCL-1 was measured. As shown in Figures 4E,F under these conditions only the concentration of 10 ng/mL exerted an inhibitory effect on the *E. coli* LPS (*p* < 0.01).

### Molecular Modeling of *Bradyrhizobium* Lipid A Binding to MD-2/TLR4

We performed computational studies to identify the possible binding modes and understand the dynamic behavior of *Bradyrhizobium* lipid A (Figure 1) in complex with human MD-2/TLR4. Since the experimental samples contain different derivatives, we studied both hopanoid-containing and hopanoid-free *Bradyrhizobium* lipid A (HOLA and HF-LA respectively, Figure 1). Docking results were evaluated based on the predicted binding score and on the apparent degree of similarity with *E. coli* LPS and lipid IV_A_ as known from their X-ray crystallographic structures accessible under the PDB accession codes 3FXI and 2E59, respectively. We considered both the insertion of the FA chains into the MD-2 pocket and the positioning of the disaccharide backbone. In addition, we systematically discarded the poses in which at least one of the saccharide-bearing acyl chains was rotated such as that the amide groups connecting the saccharide to the lipid chains were facing the opposite direction of the binding pocket. This orientation causes a large portion of the lipid chains to be exposed to the solvent, which we consider unlikely.

#### Docking Calculations of HF-LA and HOLA in MD-2

**Figure 5:**
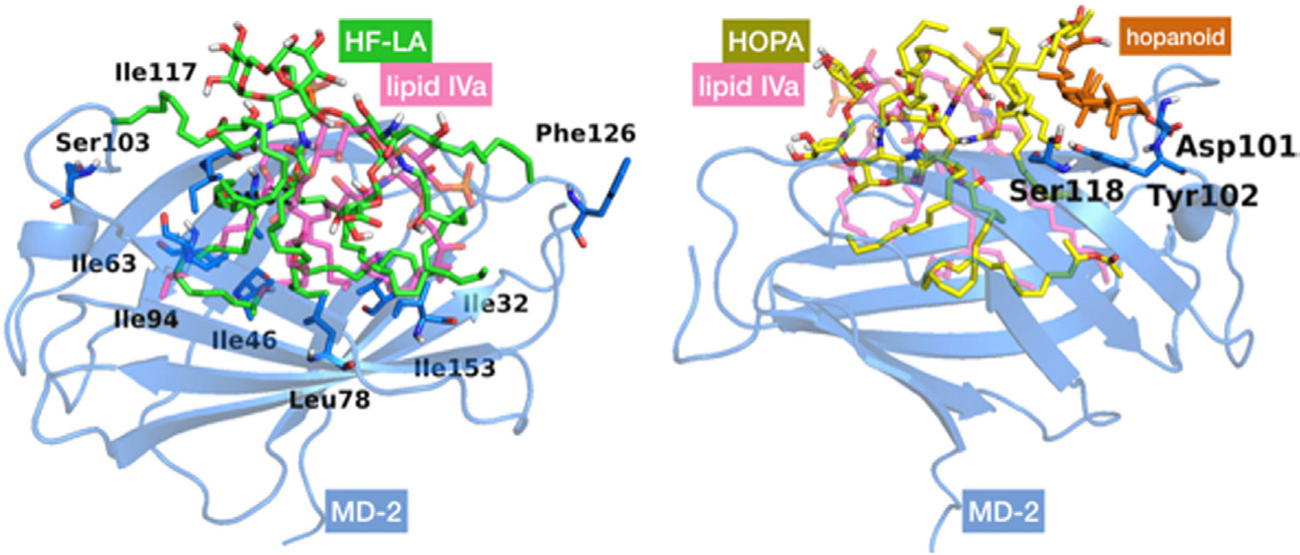
Best docked poses for HF-LA, represented in green sticks (on the left), and HOLA, in yellow sticks and the hopanoid residue in orange (on the right), inside myeloid differentiation protein-2 (MD-2) structure. Lipid IV_A_, added for comparison purposes, is depicted in pink CPK colored semi-transparent sticks. MD-2 is in blue semi-transparent cartoon. Some residues mentioned in the text are in sticks with their corresponding individual labeling.

We started by carrying out docking calculations of HF-LA and HOLA in MD-2. Plausible binding modes were obtained with most of the FA chains inserted inside the MD-2 pocket, while the sugar moieties interact at its rim. As for the interactions, in the case of HF-LA, the two VLCFA are often fully accommodated inside the MD-2 cavity where they are surrounded by hydrophobic residues, such as Val24, Ile32, 46, 63, 94, 117, 153, and Leu61, 78, leaving space for only two shorter lipid chains to enter the pocket. One of the two remaining shorter chains is often directed toward Phe126. The other one is placed in a small corridor pointing at Ser103 (Figure 5).

In the case of HOLA, the hopanoid moiety lies at the rim of the MD-2 pocket close to the residues Asp101, Tyr102, and Ser118 (Figure 5) or at a completely distinct location close to residue Lys125 and Phe126. In many docked poses, the hopanoid moiety remains on a surface outside or at the rim of the MD-2 pocket. Some low-score docking poses feature the hopanoid moiety in the hydrophobic pocket, proving that it could sterically be accommodated inside MD-2. However, in these poses, the pentasaccharide backbone is accommodated further away from the binding pocket, on a loop followed by a β-sheet formed by residues 87–91 (Figure S2 in Supplementary Material). This is likely due to the steric constraints inherent to the hopanoid moiety being inserted in the pocket. In addition, it occupies a consequent volume and seems to obstruct the passage for the lipid chains, resulting in poses in which at least three acyl chains are left outside the pocket. These results suggest that the hopanoid moiety may not play a particularly important role in the effective binding of HOLA to MD-2, and thus its presence might not be necessary for *Bradyrhizobium* to exert its antagonist activity.

#### Docking Calculations and MD Simulation of *Bradyrhizobium* Lipid A Into MD-2/TLR4 Model

**Figure 6:**
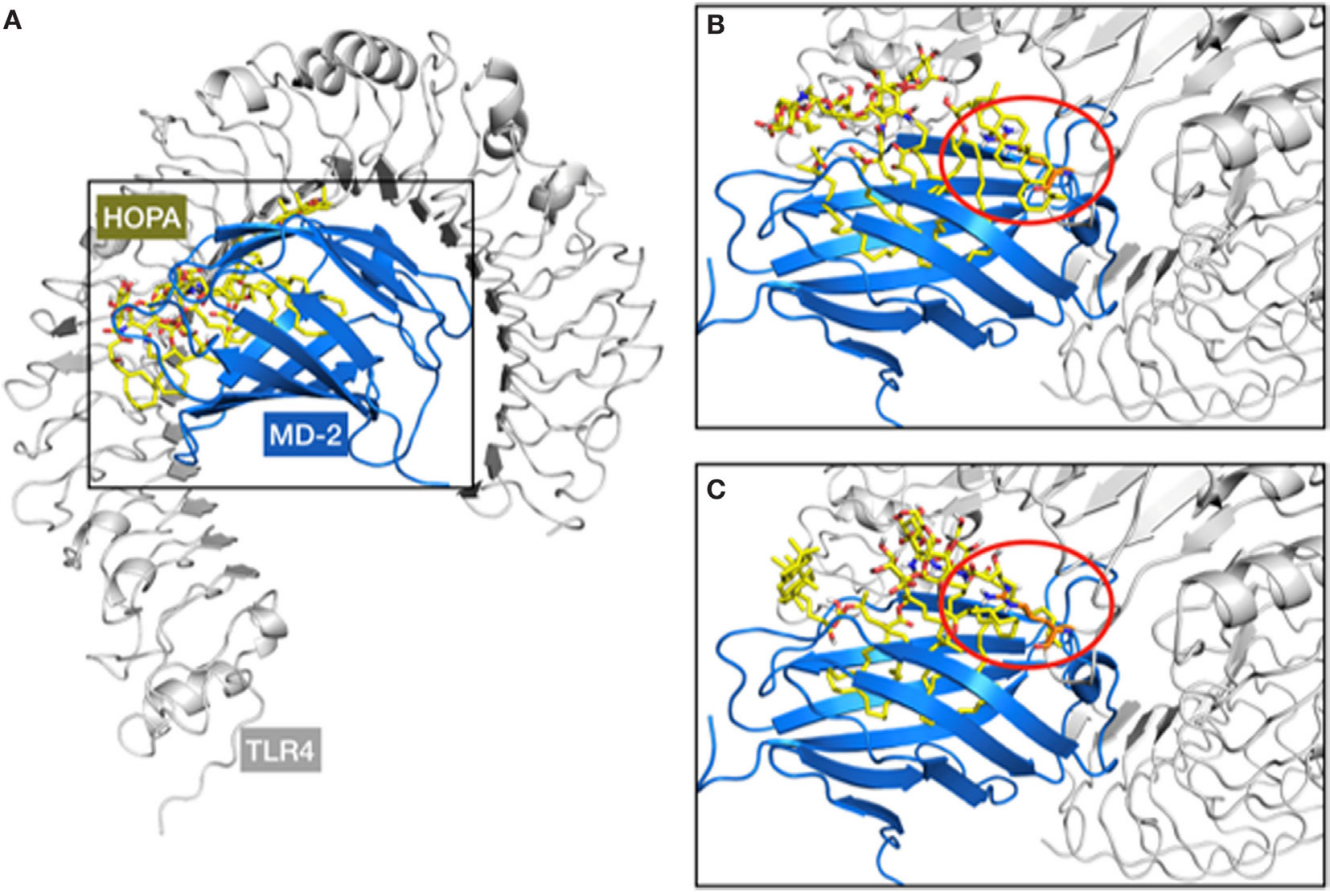
Representation of the steric clash observed over the superimposition of the myeloid differentiation protein-2 (MD-2)/HOLA complex (from docking calculations) to the MD-2/toll-like receptor 4 (TLR4) structure (from PDB ID 3FXI). A general view of the MD-2/TLR4/HOPA complex **(A)** and two examples of steric clashes are given **(B,C)**. TLR4, MD-2, and HOPA are respectively represented in gray cartoon, blue cartoon, and yellow sticks. The TLR4 protruding loop mentioned in the text is marked within the red circle, involving the hopanoid moiety **(B)** and one of the short acyl chains of HOPA **(C)**.

In a second approach, docking calculations of HOLA were performed into the MD-2/TLR4 model in the antagonist conformation reported by us somewhere else (50). Interestingly, when compared with the structure of the MD-2/TLR4 complex in the agonist conformation (PDB ID 3FXI), among the non-bonded interactions between the two proteins, a loop of TLR4, composed of amino acids 263–266, protrudes into a MD-2 channel (Figure S2 in Supplementary Material), located approximately between Asp161 and Tyr118. This protrusion is further amplified by the side chain of Arg264 that goes as far as to hover over the MD-2 hydrophobic pocket. This impingement of TLR4 over MD-2 diminishes the space available for ligand interactions in the MD-2/TLR4 complex compared with MD-2 alone. We superimposed TLR4, based on the X-ray crystallographic structure (PDB ID 3FXI), to the docking results from the MD-2-only study and noted a steric incompatibility between the TLR4 protruding loop, and either a lipid chain or the hopanoid moiety from the docked ligand (Figure 6). This observation could point to the fact that HOLA and HF-LA carry on their antagonist activity by preventing or impairing the formation of a proper MD-2/TLR4 dimer essential for TLR4 activation.

**Figure 7:**
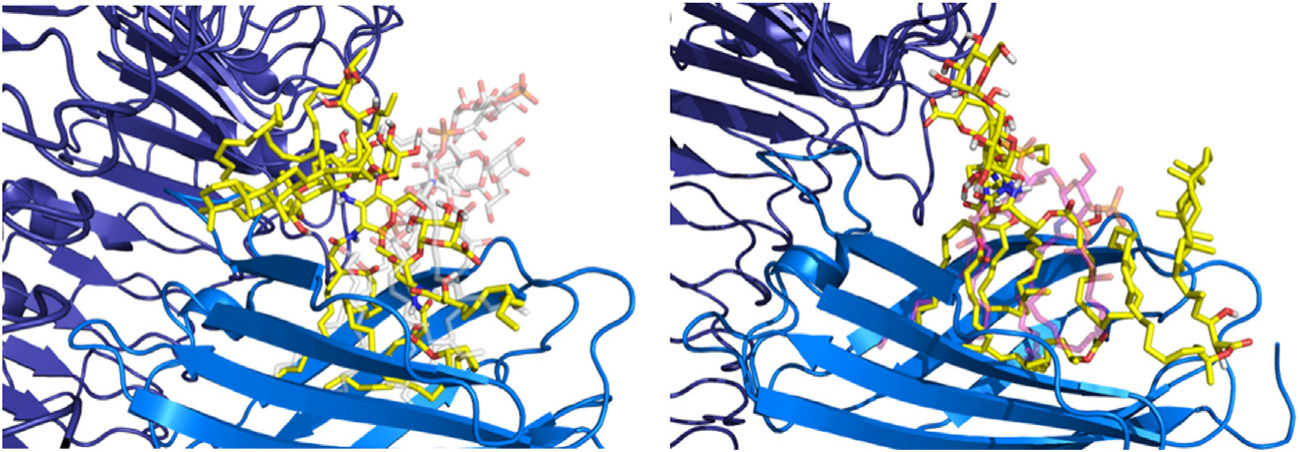
Representation of the two poses of HOPA (yellow) docked into myeloid differentiation protein-2/toll-like receptor 4 (blue/violet), *Escherichia coli* lipid A-like (on the left) and lipid IV_A_-like (on the right), selected for MD simulations.

**Figure 8:**
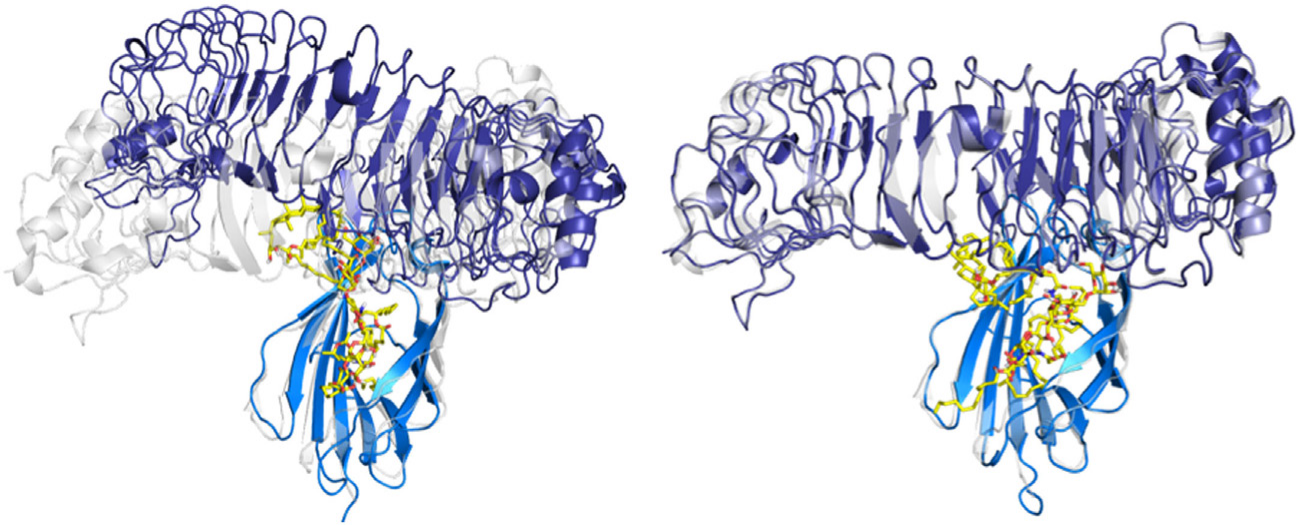
Evolution of the myeloid differentiation protein-2 (MD-2)/toll-like receptor 4 (TLR4)/HOPA complex (blue/violet/yellow) over the MD simulation. MD-2/TLR4 structure from PDB ID 3FXI, represented in semi-transparent cartoon (gray), was superimposed for comparison purposes. On the left: system at *t* = 0 ns, from the docking calculation. On the right: system at *t* = 100 ns of MD simulation.

The hydrophobic interactions taking place inside the hydrophobic pocket were essentially the same as the ones described in the case of MD-2 alone. However, the presence of TLR4, reducing the space available for ligand binding, resulted in very few poses respecting the sugar orientation criterion mentioned above. Two poses of HOLA, in good agreement with lipid IV_A_, were selected for MD simulations to further investigate interactions with the receptor and the overall stability of the MD-2/TLR4/ligand complex (Figure 7). These two poses are rotated 180° one to the other: in the first one, the HOLA lipid A is oriented as lipid IV_A_ (PDB ID 2E59) and, in the second one, it is oriented as *E. coli* LPS (PDB ID 3FXI, Figure S2 in Supplementary Material). Along the simulation time, the TLR4 presents important deviation compared to the crystal structure in relation with MD-2, as shown in the RMSD plot (Figure S4 in Supplementary Material). TLR4 displays a tendency to break apart from MD-2 indicating that the presence of the ligand destabilizes the TLR4/MD-2 complex (Figure 8).

This observation backs-up the hypothesis that *Bradyrhizobium* lipid A act as antagonists by either preventing complex formation (cf. protruding loop mentioned in the docking study above) or by disturbing the complex stability. In addition, Phe126 remains in its open conformation all along the simulation (Figure S4 in Supplementary Material), this stability was previously associated with antagonist ligands (51).

## Discussion

LPS is among the most potent pro-inflammatory compounds known, with its lipid A moiety eliciting the production of host-derived inflammatory mediators. Pyrogenicity and lethality of lipid A strictly relies on a set of structural features which includes the number and distribution of appropriately long acyl chains with respect to the glucosamine disaccharide backbone as well as the occurrence of phosphate units decorating the sugar backbone (52, 53). The possibility to prevent the detrimental effects of toxic LPS by competing lipid A derivatives with a weak or no immunostimulant power is a hot topic with an increasing appeal in several research fields (54, 55). In this context, one of the most studied is the set of synthetic analogs of lipid A of *Rhodobacter capsulatus* or *Rhodobacter sphaeroides* which are known to act as antagonists toward toxic effects of enterobacterial LPS on human cells (56–58).

Within this frame, we have recently shown that *Bradyrhizobium* lipid A present peculiar structural features comprising a pentasaccharide backbone formed by a skeleton of β-(1→6) linked 2,3-diamino-2,3-dideoxy-glucose (DAG) substituted by an α-GalA on the vicinal DAG and by an α-mannose disaccharide linked to the distal β-DAG unit (21–25); the LPS exhibits a heterogeneous blend of lipid A species, in terms of number and nature of acyl chains asymmetrically distributed on the sugar skeleton and contains VLCFA. Furthermore, as a unique peculiarity, *Bradyrhizobium* strains lipid A displays the covalent attachment of a hopanoid molecule to a VLCFA (21).

Given the not human pathogen/human associated nature of *Bradyrhizobium* strains, the unusual structural features of its lipid A, in addition to the still unresolved mechanisms at the basis of rhizobial LPS immunoactivity, prompted the investigation of the immunological properties of such a complex and novel molecule which turned out to act as a very weak agonist of murine and human immune cells. More importantly, we proved that *Bradyrhizobium* LPS is able to potently inhibit the toxic effects of *E. coli* LPS on both murine and human immune systems as a significant decrease in the cytokine release and NF-kB activation were clearly observed when cells were stimulated with both *Bradyrhizobium* LPS and lipid A and then re-stimulated with the toxic *E. coli* or *S. flexneri* LPS. We found that the inhibitory effect is especially evident at low *Bradyrhizobium* LPS concentrations. This is not surprising since this LPS is constituted by several lipid A species as mentioned above. We can hypothesize that this blend could contain one/some species provided of an inhibitory activity and others deprived of this property. By increasing the LPS concentration, the effect of these latter species prevails on that of the antagonistic species thus lowering the inhibitory capacity of this LPS. We observed the same effect with other LPS blends such as that of the intracellular *S. flexneri* (30) on *E. coli* LPS. Lipid A structures such as those of *Pseudomonas aeruginosa, Bordetella pertussis, Leptospira interrogans*, and *Neisseria meningiditis* are differently recognized by mouse and human TLR4 (48, 49). The acylation degree, the phosphate presence/absence or substitution and other still unknown molecular features of lipid A underline the differential recognition by the human and murine TLR4 complex. In line with this issue, here the inhibitory activity of *Bradyrhizobium* LPS seems to be more evident when tested on human TLR4 than on murine TLR4 (see Figures 2A,E) in this way stressing this difference. Furthermore, we also showed that *Bradyrhizobium* lipid A acts exclusively through TLR4 and do not activate TLR2 at all, in accordance with data from other lipid A strains (59). These immunological properties, as stated above, can be correlated to the peculiar structure of rhizobial LPS which showed uncommon sugar backbone, lacking phosphate decoration and presenting, among the others, VLCFA with a chain length ranging from 26 to 32 carbon atoms. In support to the hypothesis that VLCFA are involved in the potent TLR4 inhibitory activity observed for some rhizobial LPSs, a recent study showed that the human pathogen *Bartonella quintana*, expressing a penta-acylated lipid A decorated by one VLCFA [namely 26:0 (25-OH)], was able to potently block TLR4 activation by rapidly and protractedly binding the receptor complex (46). Interestingly, other intracellular pathogens belonging to *Brucella* (60, 61) and *Legionella* (62, 63) species and phylogenetically related opportunistic bacteria like *Ochrobactrum* contain VLCFA in their lipid A; in all these species, the low immunopotential of their lipid A LPS likely favors the escape from the innate immune system and the intracellular entry, as already demonstrated for other intracellular living bacteria as *Shigella* (30).

To shed light on this behavior, we investigated the molecular basis of the *Bradyrhizobium* lipid A binding to the MD-2/TLR4 complex (64). Our computational data demonstrated that the occurrence of VLCFA, likely fully accommodated inside the MD-2 cavity, may be responsible for the antagonistic properties of *Bradyrhizobium* lipid A by impairing the proper complexation of the TLR4/MD-2 dimer or potentially by destabilizing the complex itself, and, furthermore, do not point toward a primary role of the hopanoid moiety in the biological activity regarding TLR4 signaling. Our results suggest that the TLR4 signaling modulation is likely to occur by direct interaction with the TLR4/MD-2 complex, both in its hopanoid-containing and hopanoid-free forms. We indeed derived plausible binding modes of both HOLA and HF-LA to the MD-2/TLR4 system, demonstrating that *Bradyrhizobium* lipid A can act as antagonist by either preventing complex formation or by disturbing the complex stability, this accounting for the potent activity antagonizing *E. coli* LPS binding to the MD-2/TLR4 complex thus inhibiting its toxic effects. A clear understanding, at atomic level, of the molecular mechanisms leading to TLR4 activation/inhibition upon binding of *Bradyrhizobium* LPS can be of help in future prediction of the immunomodulatory properties of LPSs, and consequently of their potential use in biomedical applications, based on the chemical features of the lipid domain.

In summary, our work certainly confirmed that the rhizobia LPS world is a promising source of MD-2/TLR4 immunomodulators, that can be instrumental for the rational development of endotoxin-based therapeutics and/or vaccine adjuvants. Indeed, weak agonists, as *Bradyrhizobium* LPS has shown to be, are typically desired to be used as adjuvants in vaccine production, whereas antagonists, as stated above, are being sought as inhibitors of TLR4-dependent signaling to fight against sepsis (65). This becomes even more appealing under another point of view focused on the growing body of information describing the involvement of LPS and TLR4 in development of strongly emerging pathologies such as allergies. Indeed, it has been demonstrated that exposure to high concentrations of LPS induces allergic airway inflammation symptoms and disease *via* TLR4 signaling (66, 67). On the contrary, it has been demonstrated that environmental exposure to LPS can exert a protective action against the development of atopy and asthma (10). More interestingly, allergy-protective activity by the hepta-acylated LPS from the farm environmental bacterium *Acinetobacter lwoffii* F78 has been previously described and compared to other structurally different LPSs, such as the canonical hexa-acylated LPS from *E. coli*, which showed apparently no protective properties in concentrations comparable to a farming habitat (68). It would be tempting to say that the identification of new immunomodulatory compounds with a weak immunostimulant activity on TLR4 as well as an inhibitory action of harmful LPSs might also open to prevention of such diseases caused by abnormally exacerbated reactions to environmental factors. Therefore, determining and understanding how structural features of LPSs may affect the activation/modulation of the immune response may provide the mechanism for the fine tuning of the response itself as well as new insights to immunomodulatory processes.

## Author Contributions

AS conceived the project. AS, M-LB, and SM-S analyzed literature and designed detailed research. All the authors performed their respective experiments. FL, LL-F, J-MB, AS, M-LB, and SM-S wrote the manuscript. All authors analyzed data and revised the manuscript.

## Conflict of Interest Statement

The authors declare that the research was conducted in the absence of any commercial or financial relationships that could be construed as a potential conflict of interest.
