# Supplementary material for: Bradyrhizobium Lipid A: Immunological Properties and Molecular Basis of Its Binding to the Myeloid Differentiation Protein-2/Toll-Like Receptor 4 Complex
Source: Front Immunol. 2018 Aug 14;9:1888. doi: 10.3389/fimmu.2018.01888 (PMC6102379; doi:10.3389/fimmu.2018.01888)
Supplement: Supplementary file 1 [file Data_Sheet_1.docx]

Supplementary Material

*Bradyrhizobium* lipid A: immunological properties and molecular basis of its binding to the MD-2/TLR4 complex

Luigi Lembo-Fazio^2‡^, Jean-Marc Billod^3‡^, Flaviana Di Lorenzo^1‡^, Ida Paciello^2^, Mateusz Pallach^1^, Sara Vaz Francisco^5^, Aurora Holgado^8^, Rudi Beyaert^8^, Manuel Fresno^5^, Atsushi Shimoyama^6^, Rosa Lanzetta^1^, Koichi Fukase^6^, Djamel Gully^7^, Eric Giraud^7^, Sonsoles Martín-Santamaría^3*^, Maria-Lina Bernardini^2,4*^, Alba Silipo^1*^

*** Correspondence:**

Alba Silipo [silipo@unina.it](mailto:silipo@unina.it);

Maria-Lina Bernardini [maria.bernardini@uniroma1.it](mailto:maria.bernardini@uniroma1.it);

Sonsoles Martin-Santamaria [smsantamaria@cib.csic.es](mailto:smsantamaria@cib.csic.es)

# Supplementary Data

**Supplemental methods**

**HEK 293 hTLR2 cell culture, transfection and stimulation**

HEK293 cell line, stably transfected with human TLR2 (InvivoGen) were seeded into 96-well plates at the concentration of 1 x 10^5^ cells/mL. 24 hours after seeding the cells were transiently transfected through PolyFect Transfection Reagent (Qiagen) with a reaction mix containing 150 ng of Firefly luciferase reporter constructs, pGL3.ELAM.tk (harboring NF-κB promoter sequences), and 15 ng of *Renilla* luciferase reporter plasmid, pRLTK (as an internal control). The day after the cells were incubated with different concentrations of *Bradyrhizobium* lipid A or LPS (1, 10 and 100 ng/mL), or with purified *E. coli* LPS (LPS-EB ultrapure; InvivoGen) used at the same concentrations as above or with Pam3CSK (InvivoGen) (500 ng/mL), for 6 h to analyze NF-κB activity and to measure CXCL-8 release.

***Tlr4 -/-* and *Tlr2-/-* BMDMs isolation, culture and stimulation**

*Tlr4 -/-* and *Tlr2-/-* C57BL/6 mice were from DIOMUNE. BMDMs were derived from the bone marrow cells collected from five-week old female mice, as already reported (Paciello et al., 2013). Animal studies were conducted according to protocols approved by the University of Rome La Sapienza and adhered strictly to the Italian Ministry of Health guidelines for the use and care of experimental animals. BMDMs were differentiated during 7 days in RPMI1640 (Lonza, Italy), supplemented with 10 % of heat-inactivated FBS (HycloneTM, Euroclone, Milan, Italy.), 1 % L-glutamine (Lonza, Italy), 1 % sodium pyruvate (Lonza, Italy), 1 % NEAA (Lonza, italy), 0.5 % 2-ME (Gibco, Italy), and 40 ng/mL macrophage colony-stimulating factor (M-CSF; Miltenyi Biotec). BMDMs were seeded into 24-well plates (5 x 10^5^ cells per well) and were incubated with different concentrations of *Bradyrhizobium* LPS or lipid A (10, or 100 ng/mL) or with *E. coli* LPS (100 ng/mL) or with Pam3CSK4 for 6 h. After this time the supernatants were collected and TNF-αrelease was measured through ELISA.

# Supplementary Figures

**Figure S1. Analysis of activity of *Bradyrizhobium* LPS/lipid A in HEK293 hTLR2 cell line, *Tlr4*-/- and *Tlr2*-/- BMDMs.** Activation of NF-κB (**A**) and CXCL-8 production (**B**) in HEK 293 hTLR2 stimulated with *Bradyrizhobium* LPS/lipid A at the concentration of 1, 10 and 100 mg/mL for 6 h. Commercial hexa-acylated *E. coli* LPS used at the same concentrations or a synthetic triacylated lipopeptide, PAM3CSK (500 ng/mL) were used as controls. TNF-α release in supernatants of *Tlr4*-/- **(C)** and *Tlr2*-/- BMDMs **(D)** stimulated with *Bradyrizhobium* LPS/lipid A at the concentration of 10 and 100 mg/mL for 12 h. Commercial hexa-acylated *E. coli* LPS used at the same concentrations or a synthetic triacylated lipopeptide, PAM3CSK (1 μg/mL) were used as controls. NS: not stimulated. Data are expressed as mean ± SD of three independent experiments in triplicate. **p*<0.05, ***p*<0.01, ****p*<0.001, after Student’s *t*-test.


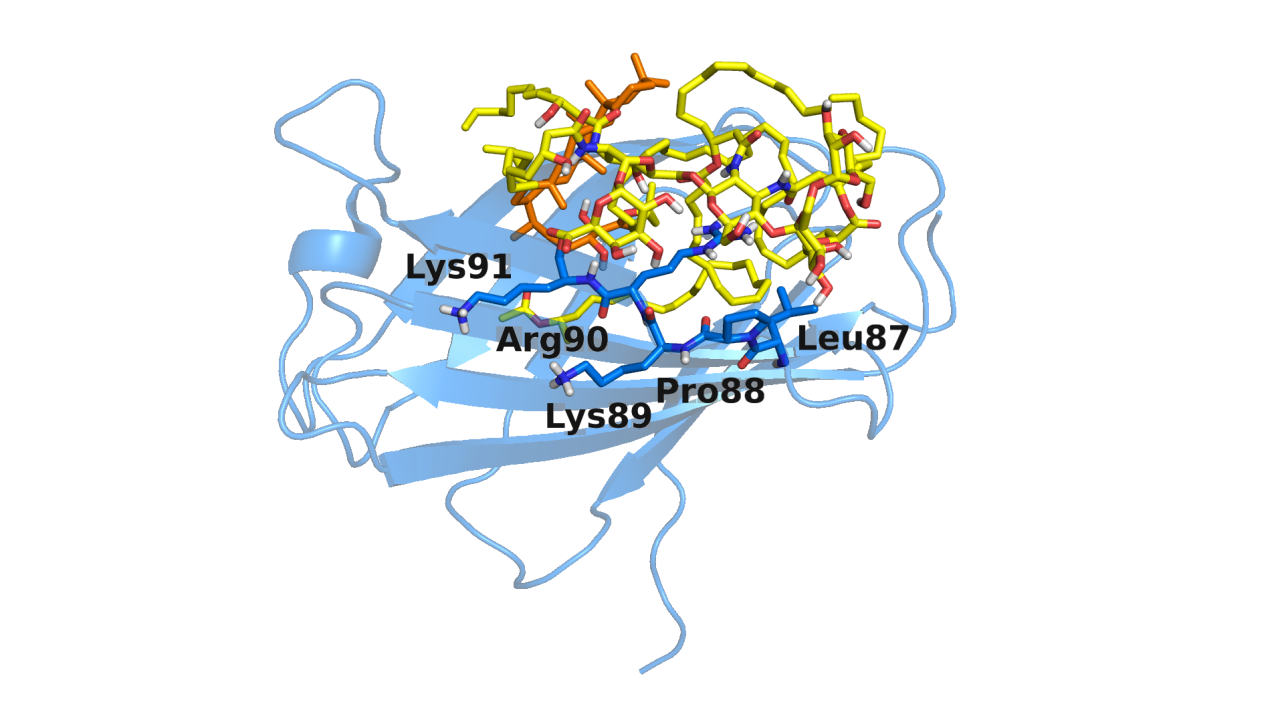
**
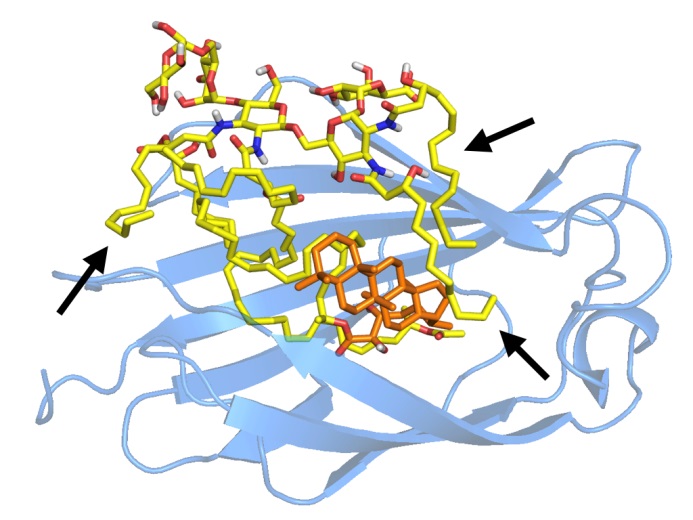
**

**Figure S2**. Example of a docked pose in which the hopanoid moiety (in orange sticks) is inserted inside the hydrophobic pocket. MD-2 (from PDB ID 2E59) is represented in semi-transparent blue cartoon and HOLA in yellow carbon and CPK colored sticks. On the left: the residues of the loop and β-sheet mentioned in the text are shown in sticks and are individually labelled. On the right: the three shorter acyl chains exposed to the solvent are indicated by black arrows.


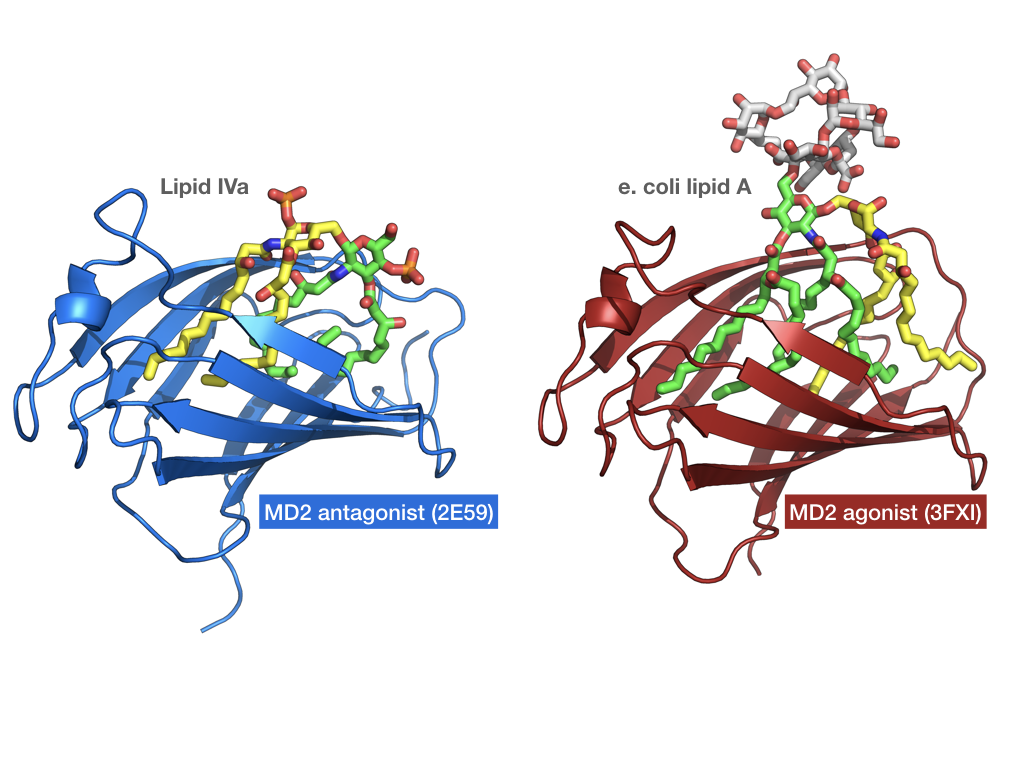


**Figure S3.** On the left: representation of type A (antagonist-like) binding mode as known from lipid IV_A_ in PDB-ID 2E59. On the right: representation of type B (agonist-like) binding mode as for *E. coli* lipid A in PDB-ID 3FXI.

| 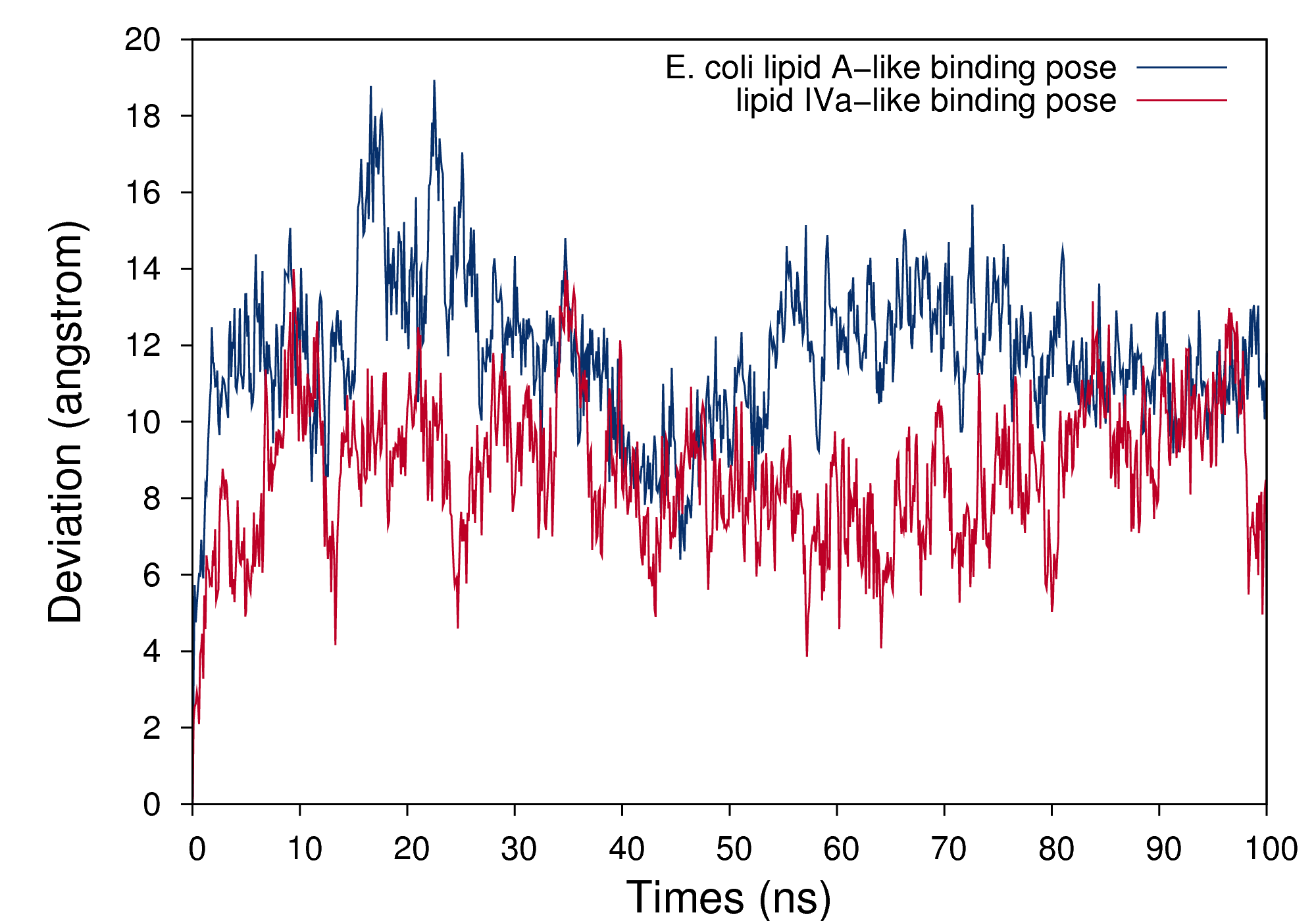 | 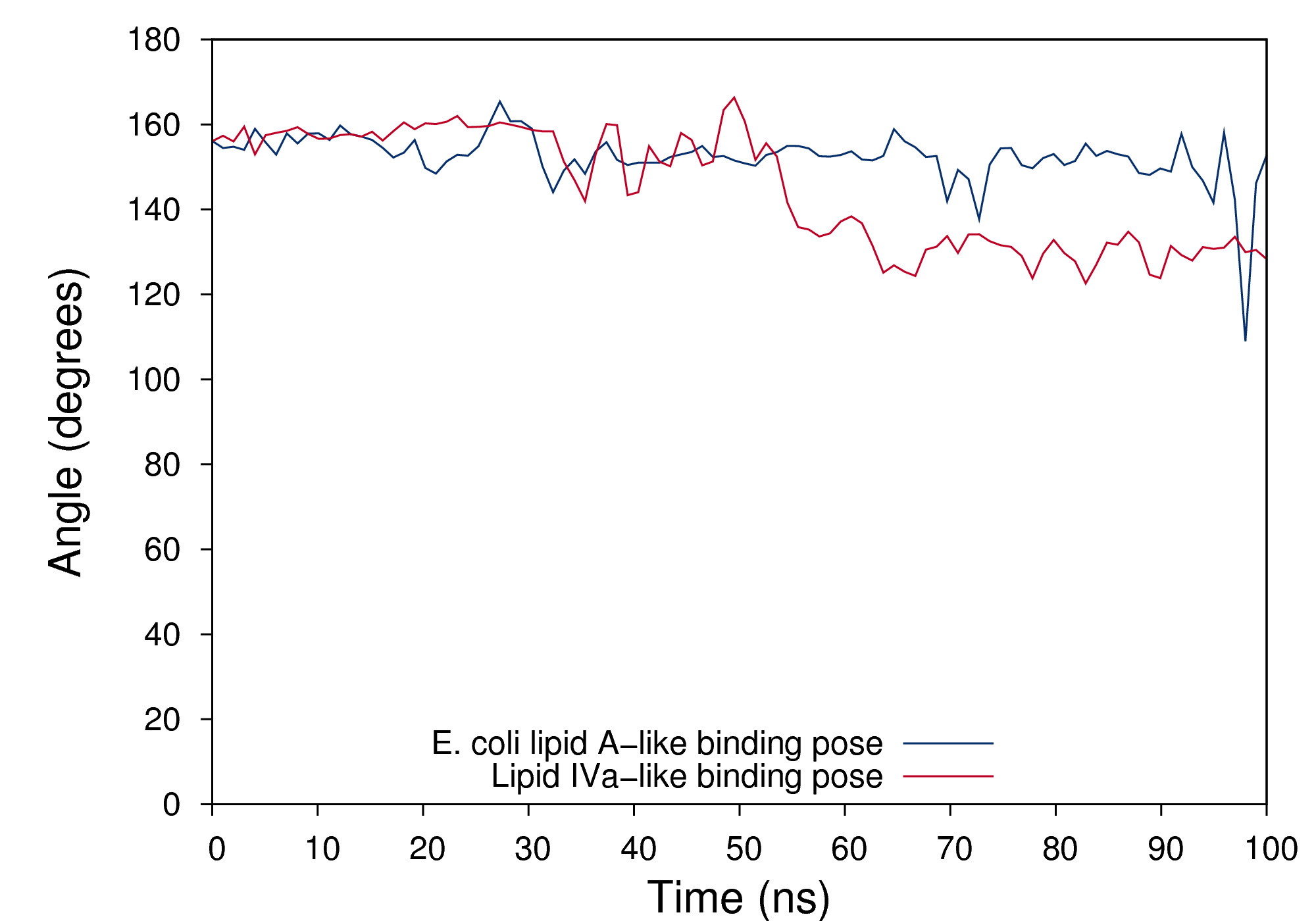 |
| --- | --- |

**Figure S4.** On the left: RMSD of TLR4 in relation to the first frame in comparison with MD-2 as the minimum fit of the system is performed on the backbone of MD-2. On the right: angle over simulation time between two arbitrarily selected vectors starting both from the α-carbon of residue Phe126 to, respectively, the zeta-carbon of the same residue and the α-carbon of residue Ser21. The angle plotted over time shows the stability of residue Phe126 during the MD simulation associated with antagonist activity of the ligand^44,45^
